# Association Between Retinal Layer Thickness and Hearing Performance in the UK Biobank: A Cross-Sectional Study

**DOI:** 10.1167/iovs.67.13.10

**Published:** 2026-11-04

**Authors:** Laura A. Meliante, Kelsey V. Stuart, Piero G. Meliante, Dun Jack Fu, Giovanni Montesano, Robert N. Luben, Mahantesh I. Biradar, Ruiqi Hu, Thomas R. P. Taylor, Danilo Iannetta, Gianluca Manni, Anthony P. Khawaja, Paul J. Foster

**Affiliations:** 1 NIHR Biomedical Research Centre, Moorfields Eye Hospital NHS Foundation Trust and UCL Institute of Ophthalmology, London, United Kingdom; 2 Department of Sense Organs, Sapienza University of Rome, Rome, Italy; 3 Department of Neuroscience, Mental Health and Sensory Organs (N.E.S.M.O.S.), Faculty of Medicine and Psychology, “Sapienza” University, Rome, Italy; 4 IRCCS – Fondazione Bietti, Rome, Italy; 5 Centre for Population Health Research and Implementation (CPHRI), SingHealth Regional Health System, Connection One, Bukit Timah Road, Singapore, Singapore; 6 Singapore National Eye Centre, 11 Third Hospital Avenue, Singapore, Singapore; 7 Health Services and Systems Research, Duke–NUS Medical School, Singapore, Singapore

**Keywords:** aging, neurodegeneration, UK Biobank, hearing loss, central retinal thickness

## Abstract

**Purpose:**

Hearing and vision loss are established risk factors for dementia, with growing evidence of shared pathogenic mechanisms. We investigated the association between hearing performance and retinal layer thickness in the UK Biobank.

**Methods:**

This cross-sectional study included 32,613 participants (mean age, 56.8 ± 8.14 years; 58.1% women) with bilateral speech reception threshold (SRT) values and optical coherence tomography–derived retinal layer measures. Multivariable linear regression models were fitted with retinal layer thickness as the outcome and the SRT as main exposure, adjusting for age, sex, ethnicity, spherical equivalent, visit type, diabetes, alcohol intake, smoking status, body mass index, education, Townsend deprivation index, and air pollution exposure. Age-equivalent differences were computed as the ratio of the SRT regression coefficient to the age coefficient.

**Results:**

Each 1-dB higher SRT (worse hearing) was associated with a thinner macula (−0.20 µm; *P* = 2.5 × 10⁻^5^), ganglion cell–inner plexiform layer (GC–IPL) (−0.09 µm; *P* = 1.1 × 10⁻^5^), inner nuclear layer (INL; −0.03 µm; *P* = 9.7 × 10⁻⁴), INL−external limiting membrane (INL–ELM; −0.0 7 µm; *P* = 3.2 × 10⁻^3^), and INL–retinal pigment epithelium (RPE) (−0.07 µm; *P* = 8.9 × 10⁻^3^) complexes. No associations were observed for retinal nerve fiber layer (RNFL), RPE, or photoreceptor layers. Participants with impaired hearing (SRT > −5.5 dB; *n* = 5,475) had a significantly thinner GC–IPL, INL, INL–ELM, INL–RPE, and total macula thickness compared with those with normal hearing (*n* = 27,138), with the greatest differences in the INL–ELM (−0.24 µm; 11.7 age-equivalent years) and INL–RPE (−0.30 µm; 6.4 years).

**Conclusions:**

Poorer hearing was independently associated with thinner middle and inner retinal layers. These findings may suggest shared neural vulnerability across auditory and visual systems, highlighting their potential as indicators of broader neural health.

Hearing impairment is the most common sensory deficit worldwide, with more than 1.5 billion people experiencing some degree of hearing loss (HL), including 430 million who require rehabilitation for disabling HL.^1^^–^^3^ Sensorineural HL (SNHL) refers to hearing impairment secondary to cochlear abnormalities and/or damage to the cochlear nerve or to the central auditory pathways, accounting for approximately 90% of all HL.^4^^,^^5^ Beyond its direct impact on social interactions and quality of life, SNHL has been linked to broader neurobiological changes leading to cognitive decline, including altered cortical activation patterns and accelerated brain atrophy.^6^^,^^7^ Age-related HL has also been associated with cognitive impairment and dementia, although the mechanisms underlying these associations remain incompletely understood.^7^ In the visual system, thinning of the retinal layers reflects structural ocular neuroaxonal loss and has also emerged as a sensitive structural marker of central nervous system neuroaxonal integrity.^8^ Retinal neurodegeneration has been associated with an increased risk of developing dementia, including Alzheimer's disease.^9^ In large population-based optical coherence tomography (OCT) studies, a thinner retinal nerve fiber layer (RNFL) has been associated with worse cognitive performance and future cognitive decline.^10^ Moreover, longitudinal studies on older adults have linked RNFL thinning with brain atrophy and episodic memory decline.^11^ These findings support the concept of the retina as a window to the brain, highlighting the potential of retinal imaging as a noninvasive, predictive biomarker of neurodegenerative processes and cognitive deterioration.^9^^,^^12^^,^^13^

Both HL and vision impairment have been identified as major modifiable risk factors for dementia.^14^ In the UK Biobank, objectively measured hearing impairment, visual impairment, and dual sensory impairment were each associated with an increased risk of incident dementia, with the highest risk estimate observed among individuals with dual sensory impairment.^15^

This association is further supported by a recent systematic review and meta-analysis that reported that dual sensory impairment was associated with an increased incidence of dementia across observational studies.^16^ Clinical studies in older adults have reported associations between hearing impairment and visual dysfunction, suggesting that auditory and visual deficits may co-occur beyond the effects of aging alone.^17^ A potential biological link between auditory and retinal health may be mediated, at least in part, by vascular dysfunction. Population-based studies have reported associations between retinal microvascular abnormalities and age-related HL, suggesting that microvascular pathology may contribute to vulnerability across sensory systems.^18^^,^^19^ Glaucoma, characterized by typical thinning of inner retinal layers, has been associated with HL, especially SNHL, supporting the hypothesis that common pathogenic mechanisms may underlie both ocular and auditory impairments.^20^ Together, these observations support the hypothesis that hearing performance and retinal structure may capture overlapping aspects of neural and vascular vulnerability.

Despite this plausible biological overlap, few studies have directly examined the relationship between hearing impairment and retinal structure.^21^^,^^22^ Existing reports have been limited to small clinical cohorts or have focused primarily on the RNFL.^21^^,^^22^ At the population level, it remains unclear whether HL is associated with retinal microanatomy, whether specific retinal layers are affected, and how large these effects are relative to normal aging.

To address these gaps, we analyzed data from the UK Biobank to investigate the association between hearing performance and OCT-derived retinal layer thickness across multiple retinal layers. We hypothesized that poorer hearing performance would be associated with thinner inner and middle retinal layers, consistent with shared neural and/or vascular vulnerability across sensory systems.

## Methods

### Study Population

This cross-sectional analysis was conducted using data from the UK Biobank, a large-scale, population-based cohort comprising more than 500,000 participants aged 37 to 73 years and recruited between 2006 and 2010. Extensive baseline assessments included lifestyle, health, physical, and biological measurements, with ongoing follow-up through repeat assessments and linkage to national health records.^23^^,^^24^ A dedicated ophthalmic substudy (2009–2010) incorporated ocular imaging with OCT.^25^

The UK Biobank received approval from the National Health Service Northwest Multicentre Research Ethics Committee (reference 11/NW/0382) and operates under the principles of the Declaration of Helsinki. All participants provided electronic written informed consent. Access to the data was obtained under UK Biobank application number 36741. Protocols are available at https://www.ukbiobank.ac.uk.

For the present study, we included participants with available data on both hearing and macular OCT.

### Hearing Assessment

Hearing was evaluated with the Digit Triplet Test (DTT), an automated measure of speech-in-noise perception. The speech reception threshold (SRT), expressed in decibels signal-to-noise ratio, is defined as the signal-to-noise ratio level at which the participant correctly identifies 50% of the digit triplets. The DTT has been shown to correlate strongly with pure-tone audiometric thresholds and is regarded as a valid measure of hearing impairment.^26^ Lower (more negative) SRT values indicate a better hearing performance.^26^^–^^28^ For each participant, the SRT values were obtained for both ears, and the average across ears was used in these analyses. We excluded subjects with conditions potentially confounding DTT performance, including hearing disorders other than SNHL, prior ear surgery, ototoxic medication use, noise exposure, and neurologic or psychiatric disorders that could impair auditory perception or cognitive processing during the DTT. Participants with extreme SRT values (>5 dB) were removed as outliers. Hearing performance was analyzed both continuously and categorically. Following UK Biobank cutoffs, hearing was classified as normal (≤−5.5 dB), insufficient (−5.5 to −3.5 dB), and poor (≥−3.5 dB).^28^^–^^30^ For the main analysis, the insufficient and poor categories were combined into an impaired hearing group to ensure statistical stability, given the limited size of the poor hearing group. Further details on hearing assessment, categorization, and exclusion criteria are provided in Supplementary Methods and Supplementary Tables S1, S2, and S3.

### Retinal Thickness Assessment

Macular OCT imaging was performed in UK Biobank using the Topcon 3D OCT-1000 Mk2 device (Topcon, Tokyo, Japan), with 6 × 6 mm² macular cube scans (128 B-scans, 512 A-scans per B-scan). Automated segmentation (Topcon Advanced Boundary Segmentation) was used to derive nine retinal layer thickness measures: RNFL, ganglion cell–inner plexiform layer (GC–IPL), inner nuclear layer (INL), INL–external limiting membrane complex (INL–ELM), INL–retinal pigment epithelium (RPE) complex, ELM–inner segment/outer segment junction complex (ELM–ISOS), ISOS–RPE complex, RPE layer, and total macular thickness.^31^^,^^32^ The left and right eye measurements were averaged for each participant. OCT values were paired with SRT data from the corresponding visit. Only participants with complete measurements for all OCT layers at the selected visit were retained. The OCT dataset used for this analysis was derived from UK Biobank OCT data after predefined quality-control procedures. Scans with an image quality score of less than 45, spherical equivalent (SE) outside ±6 diopters, poor scan-level quality metrics, or extreme retinal thickness values outside the 1st to 99th percentile range were excluded. Further details on OCT acquisition, quality control, and retinal layer definitions are provided in the Supplementary Methods, with segmentation boundaries illustrated in Supplementary Fig. S1 and layer definitions summarized in Supplementary Table S4.

### Visual Acuity (VA) Assessment

Distance VA was expressed in logMAR units. Monocular VA measurements were obtained separately for the left and right eyes and were averaged to derive a binocular VA estimate for each participant. Further details on VA derivation and data handling are provided in the Supplementary Methods.

### Assessment of Covariables

To minimize confounding, we adjusted our models for a variety of demographic, socioeconomic, lifestyle, and health-related variables. These included age, sex, ethnicity, visit type (either baseline or repeat), SE, education (quantified as age at which subjects completed full-time education), Townsend deprivation index, air pollution exposure (quantified as annual average concentrations of fine particulate matter (PM_2.5_)), diabetes, body mass index (BMI), smoking status, systemic systolic blood pressure (SBP), and alcohol consumption.^33^^,^^34^ Education was included as a proxy for socioeconomic and cognitive factors that may influence DTT performance and ocular/retinal health, consistent with previous UK Biobank analyses of OCT-derived retinal thickness.^35^^,^^36^ A detailed description of covariate definitions and derivations is provided in the Supplementary Methods.

### Statistical Analysis

We examined the association between hearing function and retinal thickness using multivariable linear regression models. Each retinal layer was modeled as the dependent variable and analyzed as a separate outcome, with the SRT as the main exposure. Two covariate adjustment models were defined a priori and applied across the analyses: model 1, which was adjusted for fixed covariables, including age, sex, SE, ethnicity, and visit type; and model 2, which was the maximally adjusted model and additionally included education, Townsend deprivation index, fine PM_2.5_ exposure, alcohol intake, diabetes, SBP, smoking status, and BMI.

These adjustment models were then applied across three complementary modelling strategies:

• Native units model: SRT (dB) and retinal thickness (µm) were analyzed in their original scales using both model 1 and model 2.
• Standardized model: SRT and retinal thickness values were standardized (*z*-scores) to allow the comparison of effect sizes across retinal layers using model 2.
• Categorical model: Hearing performance was categorized according to UK Biobank thresholds as normal (≤−5.5 dB) and impaired (comprising insufficient [−5.5 to −3.5 dB] and poor [≥−3.5 dB]). Impaired hearing was compared with normal hearing using model 2.

To aid the interpretation of effect sizes, regression coefficients for SRT were also expressed as age-equivalent changes. This transformation was applied by dividing the regression coefficient of SRT (continuous or categorical) on retinal thickness by the corresponding coefficient of one year of chronological age.

The analysis was cross-sectional and did not use repeated measures. For each participant, we retained a single pair of SRT and OCT measurements from the same visit: baseline when available; otherwise, data from the earliest follow-up visit with complete OCT layer segmentation and SRT data were used.

Effect modification by age was evaluated using two complementary approaches within model 2 (maximally adjusted). First, we fitted the SRT × age interaction terms, with age modeled continuously, to formally test whether the association between the SRT and retinal thickness varied with age. Second, to provide age-specific estimates while preserving statistical power, we repeated model 2 after stratifying participants according to the cohort age distribution, comparing the lower two age quartiles (40–58 years) with the upper two age quartiles (59–75 years). Full details are provided in the Supplementary Methods.

The association between hearing performance and VA was analyzed using multivariable linear regression, with VA (logMAR) as the dependent variable with hearing status (impaired vs. normal) as the main exposure. The same covariate set used in model 2 was applied.

Using model 2, we performed three sensitivity analyses: including participants with extreme SRT values (>5 dB); repeating the categorical analyses using the original three hearing categories (normal, insufficient, poor); and excluding participants with AMD, glaucoma, diabetic retinopathy, or other retinal vascular disease.

Results are presented as regression coefficients (β) with 95% confidence intervals. As separate regression models were fitted for each of the nine retinal layers, Bonferroni correction was applied to account for multiple comparisons, with statistical significance threshold set at a *P* value of less than 0.0056. Analyses were performed in R (version 4.4.2; The R Foundation for Statistical Computing, Vienna, Austria) using RStudio (version 2024.12.0, Posit Software, PBC).

Full details of model specification, sensitivity analyses, and implementation are provided in the Supplementary Methods.

## Results

### Participant Characteristics

A total of 32,613 UK Biobank participants were taken forward for analysis (Fig. 1). Their mean age was 56.8 ± 8.1 years; 18,962 (58.1%) were women and 13,651 (41.9%) were men. A total of 2.5% of participants self-identified as Asian, 2.2% as Black, 93.1% as White, and 1.8% as other or multiple ethnicities. All participants had complete hearing data and OCT-derived retinal layer thickness measurements. Hearing performance was classified as normal in 83.2% of participants, insufficient in 14.9%, and poor in 1.9%. Table 1 summarizes the demographic and clinical characteristics of the study cohort. The mean OCT-derived retinal thickness values are reported in Supplementary Table S5.

**Figure 1.**
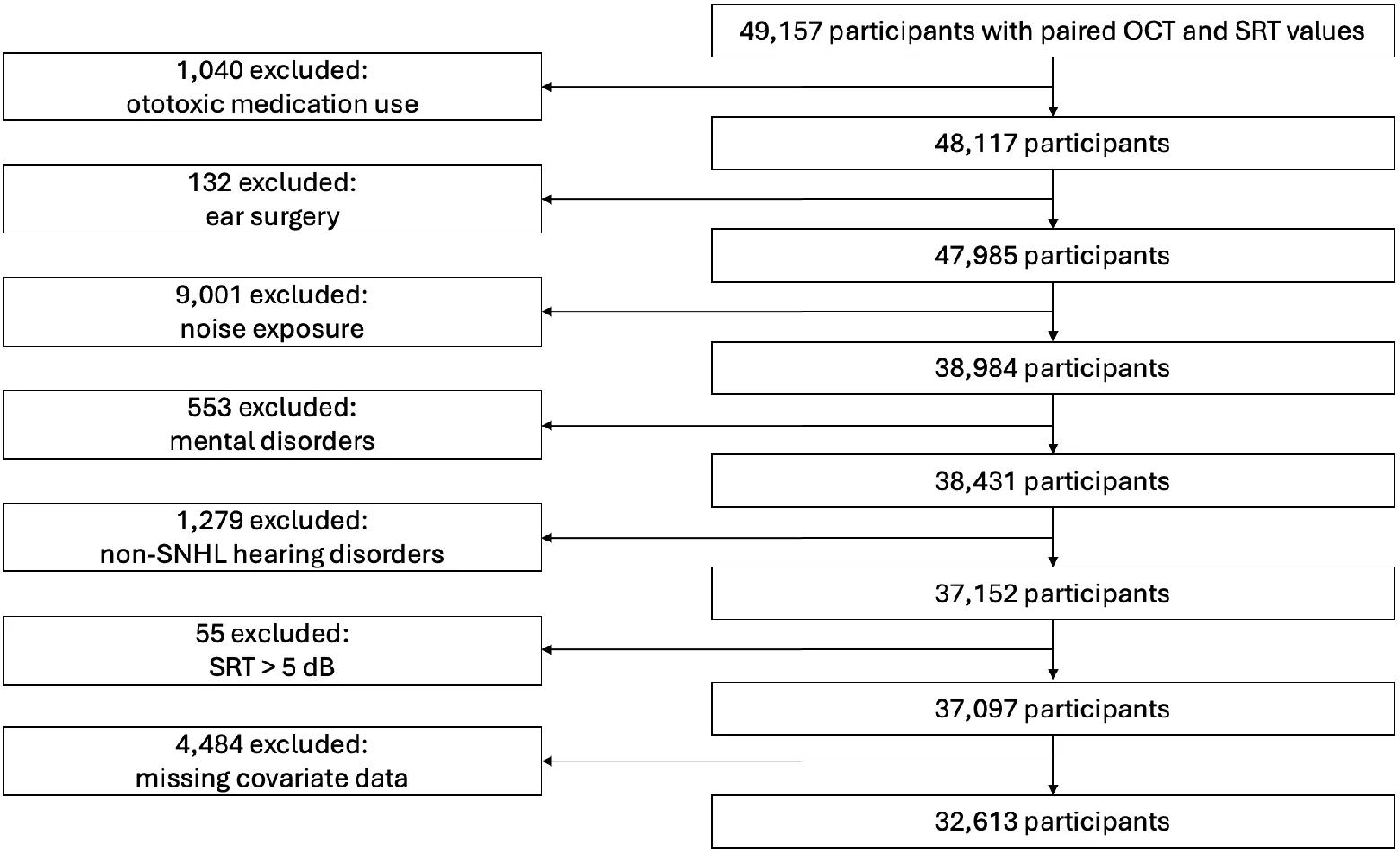
Flowchart outlining the derivation of the analytical cohort. Participants were selected from those with paired SRT values and OCT data passing predefined quality-control criteria with complete retinal layer segmentation; hearing-related exclusions and covariate completeness criteria were then applied as shown.

**Table 1.** Characteristics of Eligible UK Biobank Participants

Characteristics	Values
Age (years)	56.8 ± 8.1
Sex	
Women	18,962 (58.1)
Men	13,651 (41.9)
Ethnicity group	
White	30,353 (93.1)
Asian	829 (2.5)
Black	704 (2.2)
Mixed/other	596 (1.8)
Unknown	131 (0.4)
Age completed full time education (years)	18.6 ± 2.3
Townsend deprivation index	−1.4 ± 2.8
Alcohol intake (g per week)	111.01 ± 110.3
Pollution (PM_2.5_)	9.9 ± 0.9
Spherical equivalent	−0.1 ± 2.0
Systolic blood pressure	136.6 ± 18.4
Body mass index	26.9 ± 4.52
Diabetes	
No	30,367 (93.1)
Yes	2246 (6.9)
Smoking status	
Never	18,784 (57.6)
Previous	11,276 (34.6)
Current	2,553 (7.8)
SRT (dB)	−6.8 ± 1.4
Hearing group	
Normal	27,138 (83.2)
Insufficient	4,858 (14.9)
Poor	617 (1.9)

Values are mean ± SD or number (%).

### Association Between the SRT and Retinal Layer Thickness

#### Model Using Native Units

Higher SRT values (in dB)—indicating a poorer hearing performance—were significantly associated with reduced thickness in multiple retinal layers (in microns) in model 2 (maximally adjusted).

After Bonferroni correction (*P* < 0.0056), the strongest and most consistent associations were observed for the GC–IPL complex (β = −0.09 µm per dB increase in SRT; *P* = 1.1 × 10⁻⁵) and total macular thickness (β = −0.20 µm; *P* = 2.5 × 10⁻⁵). Significant associations were also present for the INL (*P* = 9.7 × 10⁻⁴) and the INL–ELM complex (*P* = 3.2 × 10⁻³). When expressed in terms of age-equivalent differences, a 1-dB increase in the SRT corresponded to approximately 0.6 years of aging for the GC–IPL complex, 0.7 for the INL, 0.8 for total macular thickness, and 3.2 for the INL–ELM complex. No significant associations were detected for the RNFL, ELM–ISOS complex, or RPE; the ISOS–RPE layers showed only a modest effect in model 1 that was not confirmed in model 2, while the INL–RPE complex did not survive Bonferroni correction.

These findings suggest that poorer hearing is selectively associated with thinner inner and middle retinal layers—particularly the GC–IPL complex, INL, and the INL–ELM complex (comprising the outer nuclear layer (ONL), outer plexiform layer (OPL), and Henle fiber layer)—while outer retinal structures such as the photoreceptors and RPE appear to be unaffected. Detailed results for all retinal layers are reported in Table 2.

**Table 2.** Association Between the SRT and Retinal Layer Thickness in the Core and Fully Adjusted Multivariable Linear Regression Models

Retinal Layer (µm)	Model	**β** (95% CI) Per 1 dB SRT	*P* Value	Age-Equivalent Difference (Years)
RNFL	1.	−0.02 (−0.05 to 0.01)	0.32	−0.44
	2.	−0.01 (−0.04 to +0.02)	0.53	−0.31
GC–IPL	1.	−0.09 (−0.13 to−0.05)	3.9 × 10⁻⁶*	−0.63
	2.	−0.086 (−0.12 to−0.048)	1.1 × 10⁻⁵*	−0.62
INL	1.	−0.025 (−0.041 to−0.0093)	1.9 × 10⁻³*	−0.72
	2.	−0.027 (−0.043 to−0.011)	9.7 × 10⁻⁴*	−0.71
INL–ELM	1.	−0.080 (−0.12 to−0.036)	3.6 × 10⁻⁴*	−3.9
	2.	−0.066 (−0.11 to−0.022)	3.2 × 10⁻³*	−3.3
INL–RPE	1.	−0.11 (−0.16 to−0.054)	8.4 × 10⁻⁵*	−2.2
	2.	−0.072 (−0.13 to−0.018)	**8.9 × 10⁻³**	−1.6
ELM–ISOS	1.	0.00 (−0.01 to +0.01)	0.96	−0.01
	2.	+0.0035 (−0.0067 to +0.014)	0.50	0.12
ISOS–RPE	1.	−0.029 (−0.055 to−0.0017)	**3.7 × 10⁻²**	−5.9
	2.	−0.010 (−0.037 to +0.017)	0.46	−1.5
RPE	1.	+0.014 (−0.0062 to +0.033)	0.18	0.30
	2.	+0.014 (−0.0060 to +0.034)	0.17	0.28
Macula total	1.	−0.24 (−0.33 to−0.15)	2.4 × 10⁻⁷*	−0.92
	2.	−0.20 (−0.29 to−0.10)	2.5 × 10⁻⁵*	−0.77

CI, confidence interval.

Estimates represent the change in retinal thickness (in microns, µm) per 1-dB higher SRT (poorer hearing). Model 1 includes covariates such as age, sex, SE, ethnicity, and visit type; model 2 additionally includes modifiable independent variables—education, Townsend deprivation index, fine particulate matter exposure, alcohol intake, diabetes, systolic blood pressure, smoking, and body mass index. Age-equivalent differences in retinal thickness (in years) were calculated as the ratio between the SRT coefficient and the age coefficient within each model, β(SRT)/β(Age), providing the number of years of age corresponding with the difference in retinal thickness associated with a 1-dB higher SRT in the same model. Significant associations (*P* < 0.05) are shown in bold. *P* values below the Bonferroni-adjusted significance threshold (*P* < 0.0056) are indicated with an asterisk (*).

#### Standardized Models

Maximally adjusted models (model 2) using standardized variables (*z*-scores) allowed a comparison of the magnitude of associations across the retinal layers. After Bonferroni correction (*P* < 0.0056), a 1-SD worse SRT was associated with a significantly thinner macula (β = −0.024 SD; *P* = 2.51 × 10⁻^5^), GC–IPL complex (β = −0.020 SD; *P* = 1.08 × 10⁻^5^), INL (β = −0.018 SD; *P* = 9.73 × 10⁻⁴), and INL–ELM complex (β = −0.017 SD; *P* = 3.20 × 10⁻³). A nominal association was observed for the INL–RPE complex (β = −0.015 SD; *P* = 8.85 × 10⁻³). No significant associations were detected for the RNFL, ELM–ISOS complex, ISOS–RPE complex, or RPE. Findings are illustrated in Fig. 2 (forest plot) and detailed in Supplementary Table S6.

**Figure 2.**
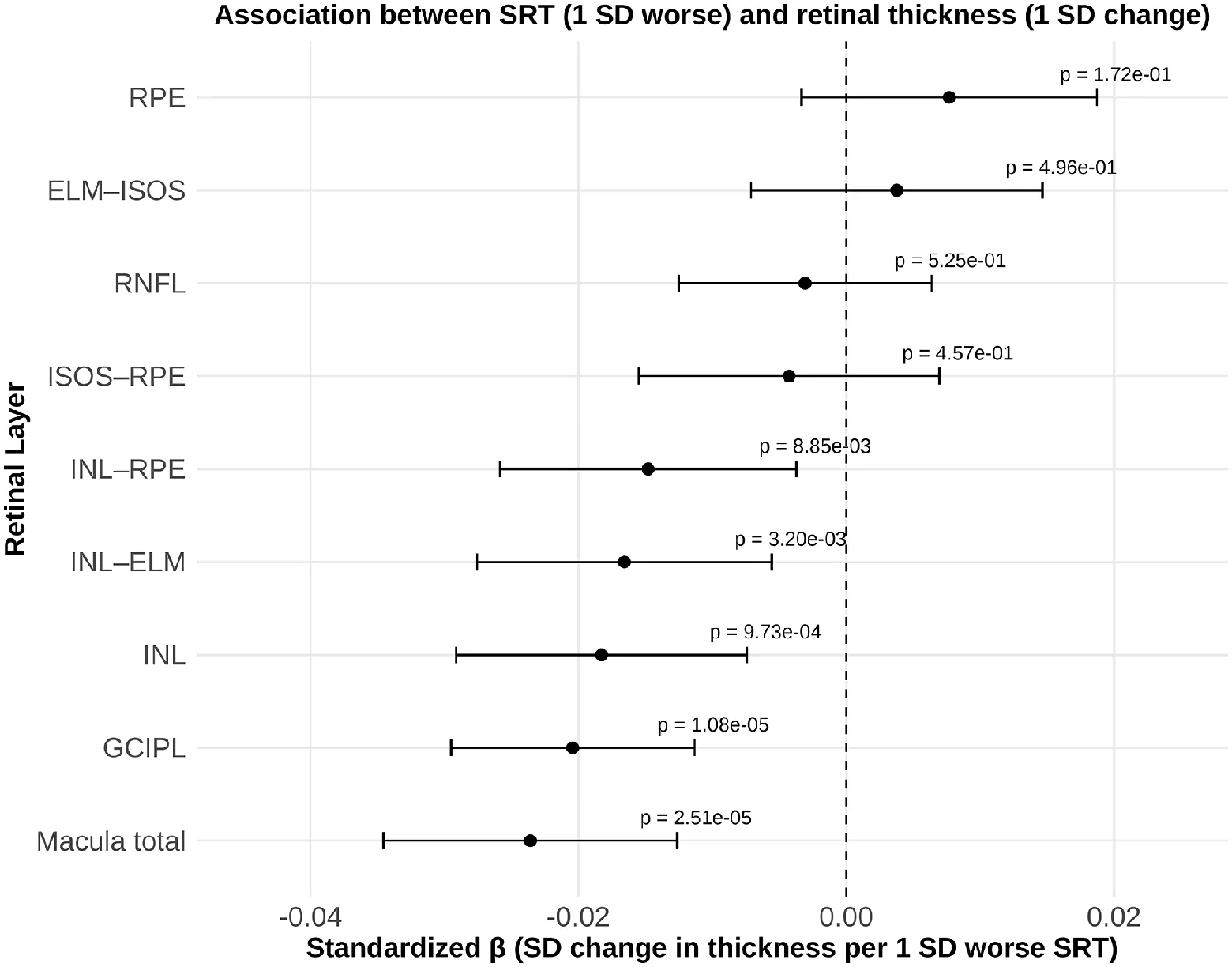
Forest plot of associations between standardized SRT (per 1 SD higher, worse hearing) and standardized retinal layer thickness (per 1 SD change). Each point shows the standardized β coefficient (SD change in retinal thickness per 1 SD worse SRT) for a given retinal layer. Horizontal lines represent 95% confidence intervals. *P* values are shown above each interval. Results are from model 2 (maximally adjusted).

#### Hearing Categories

Of the 32,613 participants included in the analysis, 27,138 (83.2%) had normal hearing and 5475 (16.8%) had impaired hearing (insufficient, *n* = 4858 [14.9%]; poor, *n* = 617 [1.9%]). The mean SRT was −7.27 ± 0.90 dB in the normal hearing group and −4.58 ± 1.21 dB in the impaired group (−4.89 ± 0.57 dB in the insufficient and −1.86 ± 1.52 dB in the poor subgroup).

Compared with participants with normal hearing, those with impaired hearing exhibited a significantly thinner GC–IPL complex (β = −0.31 µm; *P* =1.4 × 10⁻⁵), INL (β = −0.10 µm; *P* = 7.3 × 10⁻⁴), and total macula thickness (β = −0.8 µm; *P* = 4.3 × 10⁻^6^). Notably, the largest age-equivalent differences were observed for the INL–ELM complex (β = −0.24 µm, corresponding to −11.7 years) and INL–RPE complex (β = −0.30 µm, corresponding to −6.5 years). The full results are shown in Table 3.

**Table 3.** Mean Differences in Retinal Layer Thickness Comparing Impaired and Normal Hearing (Reference: Normal Hearing)

Retinal Layer	**β** (95% CI)	*P* Value	Age-Equivalent Difference (Years)
RNFL	−0.06 (−0.17 to +0.05)	0.27	−1.98
GC–IPL complex	−0.31 (−0.45 to −0.17)	1.4 × 10⁻⁵*	−2.24
INL	−0.10 (−0.16 to −0.04)	7.3 × 10⁻⁴*	−2.64
INL–ELM	−0.24 (−0.40 to −0.08)	0.004*	−11.65
INL–RPE	−0.30 (−0.49 to −0.10)	0.003*	−6.45
ELM–ISOS	−0.01 (−0.05 to 0.03)	0.626	−0.31
ISOS–RPE	−0.05 (−0.15 to 0.05)	0.335	−7.23
RPE	+0.05 (−0.02 to 0.12)	0.169	+1.05
Macula total	−0.79 (−1.12 to −0.45)	4.3 × 10⁻⁶*	−3.08

CI, confidence interval.

Maximally adjusted multivariable regression models (model 2) were analyzed, with normal hearing as the reference group. The results are expressed as regression coefficients (β, µm), 95% confidence intervals, *P* values, and years of age-equivalent difference. Negative values indicate thinner retinal layers with worse hearing. Significant associations (*P* < 0.05) are shown in bold. *P* values below the Bonferroni-adjusted significance threshold (*P* < 0.0056) are indicated with an asterisk (*).

### Age-Related Variation in the SRT–Retinal Thickness Association

Models including an SRT × Age interaction term (Supplementary Table S7) showed that the association between poorer hearing and reduced retinal thickness became progressively stronger with age for the GC–IPL complex (*P* = 0.0007), INL (*P* = 0.0024), ISOS–RPE complex (*P* = 0.041), and total macular thickness (*P* = 0.010).

The age-stratified analysis compared participants in the younger two age quartiles (40–58 years) with those in the older two age quartiles (59–75 years). Inverse associations between the SRT and retinal thickness were broadly consistent across both age groups, although they were slightly stronger in older adults for the GC–IPL complex (β = −0.060 µm per 1-dB higher SRT in those aged 40–58 years vs. −0.084 µm in those aged 59–75 years) and the INL (β = −0.017 vs. −0.027 µm, respectively). Macular thickness showed similar inverse associations in both age groups (β = −0.167 vs. −0.152 µm). The full results are shown in Supplementary Fig. S2 and Supplementary Table S8. Further details are provided in the Supplementary Results.

### VA

Participants with impaired hearing had a worse VA compared with those with normal hearing. In the maximally adjusted model, impaired hearing remained independently associated with a worse VA. Compared with participants with normal hearing, those with impaired hearing had a 0.015-logMAR higher VA (β = 0.0147; 95% confidence interval, 0.011–0.019; *P* = 1.15 × 10⁻¹³). Further details are provided in the Supplementary Results.

### Sensitivity Analyses

Results were robust to the inclusion of participants with extreme SRT values (>5 dB), with similar effect estimates and an unchanged pattern of statistical significance across the retinal layers. Of the 55 participants excluded at this step in the main cohort, 41 had complete covariate data and were included in the sensitivity analysis.

In the insufficient hearing group (*n* = 4,858), lower thickness was observed for the GC–IPL complex, INL, INL–ELM complex, INL–RPE complex, and macula, remaining significant after Bonferroni correction. In the poor hearing group (*n* = 617), associations were less stable due to the smaller sample size, and only macular thickness remained significant (β −1.36 µm; *P* = 0.003). This pattern suggests the poor hearing group lacked sufficient statistical power to yield coherent results, supporting the use of a combined impaired hearing category in the main analyses. Results for the three hearing categories are presented in Supplementary Table S9.

The results were unchanged when excluding all participants with AMD, glaucoma, diabetic retinopathy, or retinal vascular disease (*n* = 690, 534, and 196, and 116, respectively; 1389 participants with any ocular disease), confirming that underlying ocular pathology did not drive the observed associations (Supplementary Table S10). Further details on sensitivity analyses are provided in the Supplementary Results.

## Discussion

In this large, population-based study of more than 32,613 UK Biobank participants, we found that poorer hearing performance was consistently associated with a lower thickness of several macular retinal layers, particularly the GC–IPL complex, INL, total macula, INL–ELM complex, and INL–RPE complex, suggesting a selective association between hearing performance and retinal neuroaxonal structure. When expressed as age-equivalent differences, the magnitude of these associations corresponded to retinal thickness differences observed with approximately 3 to 12 years of chronological age in our model.

Previous clinical studies reported statistically significant inverse associations between HL and OCT-derived retinal thickness measures, although these studies focused primarily on RNFL-based assessments rather than a detailed evaluation of multiple retinal layers.^21^^,^^22^ By leveraging the large, well-phenotyped UK Biobank cohort, our study extends these observations and provides, to our knowledge, the first comprehensive assessment of the relationship between hearing performance and retinal thickness across the full macular depth in such a large community-based population. Our findings revealed that poorer hearing is most consistently associated with a lower thickness of the inner and middle retinal layers, while photoreceptor and RPE layers showed no significant associations. The absence of a significant association with macular RNFL could be explained by the fact that it is a relatively thin layer and may be less sensitive to subtle central neuroaxonal changes than the GC–IPL complex; moreover, the peripapillary RNFL, the measure used in most previous reports, was not available in the UK Biobank.^21^^,^^22^

Two non–mutually exclusive mechanisms may explain the association between poorer hearing performance and a lower thickness of the inner and middle retinal layers. First, this relationship may reflect shared neurovascular susceptibility across auditory and retinal tissues. The inner and mid-retinal layers, extending from the ganglion cell complex to the outer nuclear region, are supplied by the retinal circulation and contain neuronal populations—ganglion and bipolar cells—that may be more vulnerable to systemic metabolic or neurovascular influences, or to retrograde neurodegenerative processes along the visual pathway.^37^ In contrast, the photoreceptors and RPE, primarily supported by the choroidal circulation, may be less susceptible to these mechanisms.^37^ Cochlear function is also highly dependent on microvascular integrity, as the cochlear blood supply is end-arterial with limited collateral circulation, making it particularly vulnerable to vascular compromise; abnormal cochlear microcirculation has been implicated in age-related HL.^38^^,^^39^ Consistent with this hypothesis, population-based studies have reported associations between retinal microvascular abnormalities and age-related HL.^18^^,^^19^

Second, the association may reflect a shared neurosensory vulnerability affecting specialized neural tissues across sensory systems. Both the retina and the auditory pathway contain highly specialized neuronal structures characterized by a high metabolic demand.^40^^,^^41^ A recent systematic literature review showed an association between glaucoma, a progressive optic neuropathy characterized by thinning of inner retinal layers, and SNHL; no association was found with conductive HL.^20^ Consistently, studies combining neuroimaging and retinal imaging have shown that both cochlear nerve structure and RNFL thickness are reduced in individuals with hearing impairment.^22^ Together, these findings raise the possibility that hearing performance and the inner retinal structure may capture overlapping aspects of neurosensory vulnerability, although the cross-sectional nature of our data cannot establish shared or causal mechanisms. The absence of associations with photoreceptor and RPE thickness in our study further indicates that these processes are unlikely to reflect global retinal atrophy or local metabolic dysfunction. The stronger associations observed in older adults are consistent with the possibility that age-related neurodegenerative mechanisms may contribute to this sensory co-decline.

Both HL and retinal degeneration have independently been associated with dementia, although the directionality and mechanisms underlying these associations are still debated.^10^^,^^15^ Our findings suggest a possible link between these two sensory domains, adding to the evidence for a potential common degenerative pattern.^7^^,^^42^ Further studies are required to clarify whether sensory impairment represents an early marker, a contributing cause, or a downstream consequence of neurodegenerative disease.

The major strengths of this study include its large sample size, community-based design, standardized OCT imaging, and the availability of detailed covariates, which allowed for strict exclusion criteria and comprehensive adjustment to minimize confounding. By analyzing the full retinal profile available in the UK Biobank, we provide a more complete picture of the structural retinal correlates of hearing impairment. However, several limitations should be noted. First, the cross-sectional design precludes causal inference; longitudinal studies are necessary to investigate whether hearing impairment predicts progressive retinal thinning or vice versa. Second, the UK Biobank includes middle-aged and older adults, often younger than the typical age for presbycusis, which may have attenuated the observed associations. Third, the exclusion of participants with Alzheimer's disease, Parkinson's disease, and other major neurologic or psychiatric conditions strengthened the internal validity of the hearing assessment but might have limited generalizability to real-world populations in whom HL frequently co-occurs with neurodegenerative disease. Fourth, peripapillary RNFL was not captured in the UK Biobank, which may explain the lack of significant RNFL associations in our analyses compared with prior reports based primarily on peripapillary RNFL measurements.^21^^,^^22^ Additionally, automated OCT segmentation may introduce measurement error, and certain layers were only available as composite complexes rather than individually. This may have obscured more specific associations within the composite measures, particularly whether the observed INL–ELM and INL–RPE associations reflected contributions from the INL, OPL, or ONL. Fifth, hearing performance was assessed with the DTT, which captures speech-in-noise perception rather than pure-tone thresholds. Although the DTT is a validated test suitable for large-scale population studies and correlates strongly with pure-tone audiometric measures, it is not equivalent to the gold standard audiometry. DTT performance may reflect not only peripheral auditory function but also central auditory processing, attention, cognition, and language proficiency. In the UK Biobank, the DTT-derived SRT was the available objective hearing measure at scale; therefore, our findings should be interpreted as associations with functional hearing performance rather than clinically diagnosed HL. Although we excluded participants with recorded hearing disorders other than SNHL, prior ear surgery, ototoxic medication use, noise exposure, and neurologic or psychiatric conditions that could confound DTT performance, SRT cannot itself differentiate sensorineural, conductive, mixed, or central auditory causes of impaired performance. Residual misclassification therefore cannot be excluded, particularly because HL phenotyping in the UK Biobank relies partly on hospital diagnostic codes and not all participants with hearing impairment have a recorded clinical diagnosis. Consequently, some non-SNHL or reversible causes of HL—such as cerumen impaction—may not have been fully captured. Additionally, residual confounding and the predominantly White composition of the UK Biobank may limit the generalizability of our findings. Finally, axial length was not measured as a part of the standard UK Biobank ophthalmic assessment and therefore could not be included as a covariate. Although all the models were adjusted for SE and participants with an extreme refractive error outside ±6 diopters were excluded, SE may not fully capture ocular size or OCT magnification effects, particularly in pseudophakic eyes or in eyes where the refractive error is influenced by the lens status. Residual confounding related to axial length therefore cannot be excluded.

The potential clinical and public health relevance of these findings lies in their population-level magnitude and layer-specific pattern. Expressed in years of age-equivalent difference, the magnitude of retinal thickness differences associated with poorer hearing performance was substantial at the population level—reaching up to 12 years in composite inner–outer retinal measures in categorical analysis. These results suggest that hearing performance and retinal thickness may capture overlapping aspects of broader neural health. Given that both HL and visual impairment are recognized as modifiable risk factors for dementia, a lower retinal thickness may represent a structural correlate of the sensory–cognitive risk pathway.^14^ Nevertheless, given the cross-sectional design, we cannot determine whether retinal differences precede, follow, or predict the progression of hearing dysfunction. The predominance of inner and middle retinal associations, with no significant associations in the photoreceptor or RPE layers, suggests that further research should examine whether these retinal measures relate to more detailed auditory phenotypes and to a broader neurosensory or neurovascular vulnerability. In our analyses, VA was also lower in participants with impaired hearing; however, given the predominance of the inner retinal associations, further studies should examine whether poorer hearing performance is associated with other aspects of visual function mediated by inner retinal pathways, such as visual field sensitivity or contrast sensitivity, rather than VA alone. Studies incorporating pure-tone audiometry, repeated hearing assessments, clinical examination to exclude reversible or transient causes of hearing impairment, objective auditory biomarkers where available, and detailed visual function testing would help to validate and extend these findings. Older cohorts with more prevalent presbycusis and dementia would be particularly informative.

## Conclusions

Our study highlights a significant association between hearing performance and retinal structure at the population level, consistent with the hypothesis of shared neurodegenerative pathways across sensory systems. As the prevalence of dementia continues to rise worldwide due to the population aging, becoming an increasingly greater public health challenge, hearing and visual impairment are recognized risk factors for its development.^14^ Understanding the mechanisms that underlie this common vulnerability may help to clarify how sensory impairment relates to broader neural health, and possibly open the way to tackling the roots of neurodegeneration, rather than only its consequences.

## Supplementary Material

Supplement 1

Supplement 2

Supplement 3
