# Supplementary material for: Association Between Retinal Layer Thickness and Hearing Performance in the UK Biobank: A Cross-Sectional Study
Source: Invest Ophthalmol Vis Sci. 2026 Nov 4;67(13):10. doi: 10.1167/iovs.67.13.10 (PMC13743836; doi:10.1167/iovs.67.13.10)
Supplement: Supplementary file 1 [file iovs-67-13-10_s001.docx]

**Supplementary Methods**

# **Hearing Assessment**

Hearing was assessed in the UK Biobank (UKB) using the **Digit Triplet Test (DTT)**, a validated **speech-in-noise** test widely used in large-scale epidemiological studies.^1–3^ The DTT evaluates the ability to recognize speech against a background noise by presenting participants with sets of three spoken digits (e.g., 1–5–8) delivered through headphones. The signal-to-noise ratio (SNR) – the relative level of the speech signal compared to the masking noise – is adaptively adjusted across trials based on the participants’ responses. The outcome of the test is the **speech reception threshold (SRT)**, defined as the **signal-to-noise ratio (SNR)** at which 50% of the digit triplets are correctly identified.^1–3^

The **SRT** is expressed in decibels (dB). Lower (more negative) values correspond to better hearing performance, as they indicate that speech recognition is possible at more adverse SNRs. Conversely, higher (less negative) values indicate poorer hearing. ^1–3^

DTT correlates strongly with pure tone audiometric measures, with correlation coefficients (r) greater than 0.7 in most studies. This strong agreement supports the validity of DTT-derived SRT as a measure of functional hearing performance and its use in correlation-based analyses.^1–4^

In this study, we used both continuous SRT values and categorical groupings based on UK Biobank thresholds:

- **Normal hearing**: SRT ≤ –5.5 dB
- **Insufficient hearing**: –5.5 to –3.5 dB
- **Poor hearing**: SRT ≥ –3.5 dB. ^1–4^

For each participant, SRT values were available separately for left and right ear. When data from both ears were available, the average was used; if only one ear had a valid value, that measurement was retained. SRT values were paired with OCT imaging from the corresponding visit (baseline or repeat). Baseline values were prioritised when complete; otherwise, repeat-visit values were used. Outliers with implausible values (SRT > 5 dB) were excluded for the main analysis.

## Exclusion criteria

Participants with diagnostic codes consistent with SNHL were retained, including ICD-10 codes H90.3, H90.4, H90.5 and ICD-9 code 389.1 (sensorineural deafness).

To avoid inclusion of participants with conditions that could confound DTT performance, we applied the following exclusions based on **ICD-9/10 diagnostic codes, operation codes, medication data, and questionnaire data**:

1. Participants with other ear disorders or hearing conditions potentially consistent with conductive or mixed hearing loss (Supplementary Table 1).
2. **History of ear surgery** (UKB field 20004, Operation codes):
   a. 1583: Mastoidectomy/mastoid surgery
   b. 1584: Stapedectomy
   c. 1616: Tympanic membrane surgery/ear drum repair
   d. **Exposure to ototoxic drugs** (UKB field 20003; Supplementary Table 2).
3. **Noise exposure** (UKB field 4825):
   a. Excluded if reporting prolonged noisy workplace exposure (codes 11, 12, 13: >5 years, 1–5 years, or <1 year).
4. **Conditions – such as cognitive impairment or mental disorders** – which could impair performance on hearing tests and introduce bias into the assessment of sensorineural hearing loss (ICD-9/10 diagnoses; UKB fields 41270, 41271; Supplementary Table 3).
5. **Outliers in speech reception threshold (SRT):**
   a. Participants with SRT > 5 dB in both ears were excluded.
   b. For individuals with a unilateral outlier, the valid ear was retained to avoid unnecessary loss of data.

After these exclusions, participants with valid SRT data and without recorded non-SNHL hearing conditions or other factors likely to confound DTT performance were retained for analysis.

**Supplementary Table 1.** Excluded hearing-related conditions (non-SNHL) with corresponding ICD-9 and ICD-10 codes (UK Biobank fields 41270, 41271)

| **ICD System** | **Code(s)** | **Description** |
| --- | --- | --- |
| **ICD-10** | H60.0–H60.9 | Disorders of external ear (abscess, cellulitis, otitis externa, cholesteatoma, impacted cerumen, stenosis, other/unspecified) |
|  | H61.0–H61.9 | Other external ear disorders (perichondritis, disorders of pinna, cerumen, stenosis, other/unspecified) |
|  | H62.2, H62.8 | Otitis externa in mycoses / other diseases classified elsewhere |
|  | H65.0–H65.4, H65.9 | Nonsuppurative otitis media (acute/chronic serous or mucoid, other/unspecified) |
|  | H66.0–H66.4, H66.9 | Suppurative otitis media (acute/chronic/unspecified) |
|  | H68.0–H68.1, H69.0, H69.8–H69.9 | Eustachian tube disorders (salpingitis, obstruction, patulous, other/unspecified) |
|  | H70.0–H70.9 | Mastoiditis and related conditions |
|  | H71.0 | Cholesteatoma of middle ear |
|  | H72.0–H72.9 | Perforation of tympanic membrane (central, attic, marginal, other/unspecified) |
|  | H73.0–H73.9 | Myringitis and other tympanic membrane disorders |
|  | H74.0–H74.9 | Middle ear and mastoid disorders (tympanosclerosis, ossicle abnormalities, adhesive disease, polyp, other/unspecified) |
|  | H80.0–H80.9 | Otosclerosis (oval window, cochlear, other/unspecified) |
|  | H81.0, H81.2–H81.4, H81.8–H81.9 | Vestibular disorders (Ménière’s disease, neuronitis, vertigo of central origin, other/unspecified) |
|  | H82.0 | Vertiginous syndromes in diseases classified elsewhere |
|  | H83.0–H83.3, H83.8–H83.9 | Labyrinthitis, fistula, dysfunction, noise effects, other/unspecified inner ear disease |
|  | H90.0–H90.2, H90.6–H90.8 | Conductive and mixed hearing loss (bilateral, unilateral, unspecified) |
|  | H91.0, H91.2–H91.3, H91.8–H91.9 | Other specified/unspecified hearing loss, ototoxic HL, sudden idiopathic HL, deaf mutism |
|  | H92.0–H92.2 | Otalgia, otorrhoea, otorrhagia |
|  | H93.0, H93.2–H93.3, H93.8–H93.9 | Ear disorders (degenerative/vascular, auditory perception abnormalities, acoustic nerve, other/unspecified) |
|  | H94.0, H94.8 | Acoustic neuritis and other specified ear disorders in systemic disease |
|  | H95.0–H95.9 | Post-mastoidectomy and other postprocedural ear/mastoid disorders |
| **ICD-9** | 3800–3804, 3808–3809 | External ear disorders (perichondritis, otitis externa, pinna disorders, cerumen, other/unspecified) |
|  | 3810–3814, 3816, 3819 | Nonsuppurative otitis media and Eustachian tube disorders (acute/chronic, obstruction, unspecified) |
|  | 3821–3823, 3829 | Suppurative/unspecified otitis media (chronic tubotympanic, atticoantral, other/unspecified) |
|  | 3831, 3833, 3838–3839 | Mastoiditis and complications (chronic, post-mastoidectomy, other/unspecified) |
|  | 3842, 3848 | Tympanic membrane disorders (perforation, other specified) |
|  | 3850–3853, 3858 | Middle ear/mastoid disorders (tympanosclerosis, adhesive, ossicle abnormalities, cholesteatoma, other specified) |
|  | 3860–3861, 3863, 3869 | Vertiginous syndromes/vestibular disorders (Ménière’s, peripheral vertigo, labyrinthitis, unspecified) |
|  | 3872, 3879 | Otosclerosis (cochlear, unspecified) |
|  | 3886–3889 | Other ear disorders (otorrhoea, otalgia, other specified, unspecified) |
|  | 3890, 3892, 3897, 3899 | Hearing loss (conductive, mixed, deaf-mutism, unspecified) |

**Supplementary Table 2.** Medications with ototoxic potential (UK Biobank field 20003).

| **Drug Class** | **Drug** | **UKBB Code** |
| --- | --- | --- |
| **Aminoglycoside Antibiotics** | Streptomycin | 1140874098 |
|  | Gentamicin | 114087574 |
|  | Kanamycin | 1140873496 |
|  | Amikacin | 1140873588 |
|  | Tobramycin | 1140873536 |
|  | Neomycin | 1140884090 |
|  | Netilmicin | 1140873514 |
|  | Mycifradin (Neomycin-related) | 1140873512 |
|  | Nivemycin (Neomycin-related) | 1140873506 |
|  | Tobramycin (Nebcin 20mg/2ml injection) | 1140873538 |
|  | Tobramycin (Nebcin 40mg/1ml injection) | 1140873540 |
|  | Tobramycin (Nebcin 80mg/2ml injection) | 1140873542 |
| **Tetracyclines** | Tetracycline | 1140873450 |
|  | Minocycline | 1140873480 |
|  | Doxycycline | 1140873394 |
|  | Oxytetracycline | 1140873548 |
| **Polymyxins** | Polymyxin B | 1140873842 |
|  | Colistin (Colomycin 1.5 million units tab) | 1140873812 |
|  | Colistin (Colomycin 500000 units inj) | 1140873816 |
|  | Colistin (Colomycin 1 million units inj) | 1140873818 |
| **Fluoroquinolones** | Ciprofloxacin | 1140874138 |
|  | Ofloxacin | 1140874160 |
|  | Norfloxacin | 1140874178 |
| **Loop Diuretics** | Furosemide | 1140909708 |
|  | Bumetanide | 1140866280 |
| **Macrolide Antibiotics** | Erythromycin | 1140884016 |
|  | Clarithromycin | 1140883634 |
|  | Azithromycin | 1140883642 |
| **Chemotherapy Agents** | Busulphan | 1140869578 |
|  | Cyclophosphamide | 1140869604 |
|  | Methotrexate | 1140869848 |
|  | Fluorouracil | 1140869772 |
| **Antimalarials** | Quinine | 1140874420 |
|  | Chloroquine | 1140874482 |
|  | Hydroxychloroquine | 1140884308 |
| **Antiepileptics** | Vigabatrin | 1140872280 |
|  | Valproate (Sodium Valproate/Orlept) | 1140872268 |

**Supplementary Table 3.** Cognitive and neurological conditions excluded due to potential impact on Digit Triplet Test performance

| **UKBB field** | **ICD code(s)** | **Description** |
| --- | --- | --- |
| 41270 (ICD-10) | F00–F03 | Dementia (Alzheimer’s, vascular, other) |
|  | F05 | Delirium (non-substance-induced) |
|  | F06 | Organic mental disorders due to brain damage/disease |
|  | F20–F23 | Schizophrenia and related psychoses |
|  | F25 | Schizoaffective disorders |
|  | F31.0–F31.6 | Bipolar disorder, current episodes |
|  | F32.2–F32.3 | Severe depressive episode (± psychosis) |
|  | F33.2–F33.3 | Recurrent severe depression |
|  | G20 | Parkinson’s disease |
|  | G30–G32 | Alzheimer’s and other degenerative CNS diseases |
|  | G35 | Multiple sclerosis |
|  | G36–G37 | Other demyelinating CNS diseases |
|  | G40–G42 | Epilepsy and seizure disorders |
| 41271 (ICD-9) | 290–294 | Senile/organic psychoses & dementia |
|  | 295 | Schizophrenic psychoses |
|  | 296.0–296.6 | Manic-depressive psychoses |
|  | 298 | Other non-organic psychoses |
|  | 293 | Transient organic psychotic conditions |

# **Retinal thickness**

## **OCT assessment**

Spectral-domain OCT imaging was performed using the Topcon 3D OCT-1000 Mk2 (Topcon, Tokyo, Japan) under mesopic conditions, without pharmacological dilation. A 6×6 mm² macular cube scan (128 B-scans, 512 A-scans per B-scan) was acquired. To ensure data quality, all OCT scans in UK Biobank underwent centralised monitoring and grading for image quality, focus, foveal centration, and segmentation accuracy. Feedback on errors attributable to participants, operators, or equipment was provided to acquisition centres on an ongoing basis.^5^ Segmentation was performed centrally using the Topcon Advanced Boundary Segmentation (TABS) algorithm which defined nine intraretinal boundaries (Supplementary figure 1), from which thickness measures were derived and analysed (Supplementary Table 4).^5,6^

**Supplementary Figure 1.** Automated segmentation of optical coherence tomography (OCT) macular scans using the Topcon Advanced Boundary Segmentation (TABS™) algorithm, which defines nine intraretinal boundaries (yellow lines). Boundaries: 1 = ILM, 2 = NFL/GCL, 3 = GCL/IPL, 4 = IPL/INL, 5 = INL/OPL, 6 = ELM, 7 = EZ, 8 = OS/RPE, 9 = BM.
Reproduced from Keane PA et al., PLoS ONE 2016;11(8):e0164095, doi:10.1371/journal.pone.0164095, under CC-BY 4.0 license.

**Supplementary Table 4.** Retinal layers defined by UK Biobank segmentation boundaries.

| **UKB variable name** | **Boundary indices** | **Anatomical layer / description** |
| --- | --- | --- |
| RNFL | 1–2 | Retinal nerve fibre layer |
| GCIPL | 2–4 | Ganglion cell layer + inner plexiform layer |
| INL | 4–5 | Inner nuclear layer |
| INL–ELM | 5–6 | Outer plexiform layer (incl. Henle fibre layer) + outer nuclear layer |
| INL–RPE | 4–8 | Inner nuclear layer + Outer plexiform layer (incl. Henle fibre layer) + outer nuclear layer + photoreceptors (to inner RPE boundary) |
| ELM–ISOS | 6–7 | Photoreceptor inner segments, from ELM to ellipsoid zone |
| ISOS–RPE | 7–8 | Photoreceptor outer segments, from ellipsoid zone to inner Retinal pigment epithelium |
| RPE | 8–9 | Retinal pigment epithelium |
| Macula (total) | 1–8 | Full retinal thickness (RNFL to RPE, excluding RPE) |

## OCT data cleaning and quality control

OCT measurements were obtained either during baseline or during the first repeat assessment, and were subsequently processed with the following quality-control steps:

First, we excluded scans with an image quality score below 45 and participants with spherical equivalent refractive error outside ±6 diopters, as extreme refractive error can distort retinal thickness measurements. In addition, we applied distribution-based cutoffs to several scan-level quality metrics. For *ilm_indicator*, *valid_count*, and *min_motion_correlation*, values below the 20th percentile were excluded; for *max_motion_delta* and *max_motion_factor*, values above the 80th percentile were excluded. These percentile-based thresholds ensured the removal of the lowest-quality quintile of images for each metric, while retaining the majority of acceptable scans. Values beyond the 1st and 99th percentiles of each retinal thickness distribution were classified as outliers and therefore removed. For participants with data from both eyes, mean thickness values were computed; if only one eye passed quality control, the available measurement was retained.

OCT values were paired with SRT data from the corresponding visit (baseline or repeat), ensuring that hearing and retinal data were contemporaneous. Only participants with complete measurements for all OCT layers were retained for analysis. Baseline OCT values were prioritised when complete; otherwise, repeat-visit values were used.

# Visual acuity assessment

Distance visual acuity (VA) was assessed in the UK Biobank as part of the ophthalmic assessment using habitual correction. Monocular VA measurements were recorded separately for the left and right eyes using UK Biobank data fields 5208 (left eye) and 5201 (right eye), expressed in logarithm of the minimum angle of resolution (logMAR) units, with higher values indicating worse VA.

For each participant, VA measurements were aligned with the same assessment visit used for the hearing evaluation (DTT) and retinal OCT imaging. When VA data were available at both baseline and repeat assessments, measurements from the visit corresponding to the paired hearing and OCT data were used. When VA was missing at the selected visit, values from the alternative visit were used as a fallback, prioritising temporal proximity to the hearing assessment.

Monocular VA values from the left and right eyes were averaged to derive a binocular mean VA for each participant, which was used as the outcome variable in all VA analyses.

In the UK Biobank protocol, distance VA was measured under habitual correction. Participants were instructed to wear their usual distance glasses or contact lenses if required. The availability of habitual distance correction at the assessment visit as recorded separately for each eye using UK Biobank data fields **6075** (left eye) and **6074** (right eye), coded as:

- **0** = no correction required
- **1** = correction worn at the visit
- **2** = correction usually worn but unavailable at the visit (“elsewhere”)

Participants recorded as having required distance correction that was unavailable at the assessment visit (“elsewhere”) in either eye were excluded from all VA analyses. This exclusion was applied to minimise misclassification due to uncorrected refractive error, which could artificially worsen VA measurements in individuals who normally use optical correction but did not have access to it at the time of testing.

# **Assessment of covariables**

All covariables used in the analyses were ascertained at the same visit as OCT and SRT measurements (baseline or first repeat assessment, depending on availability). When values from the paired visit were missing, the corresponding measure from the alternative visit was used as fallback.

Variables were derived from UK Biobank fields as follows:

Age
Age at assessment was obtained from UK Biobank field 21003 (age when attended assessment centre). For each participant, age was aligned with the visit from which paired OCT and SRT data were taken. Specifically, if paired data were from the baseline visit, we used 21003_0_0; if paired data were from the repeat visit, we used 21003_1_0.

Sex
Biological sex was derived from UK Biobank field 31. Values were coded as Female (0) and Male (1).

Ethnicity
Ethnic background was obtained from UK Biobank field 21000. We preferentially used the baseline response (21000_0_0), falling back to repeat assessment (21000_1_0) if baseline was missing. Ethnic categories were grouped as follows:

- White (codes 1001, 1002, 1003)
- Black (codes 4001, 4002, 4003)
- Asian (codes 3001, 3002, 3003, 3004, 5)
- Mixed / Other (codes 2001, 2002, 2003, 2004, 6)

Participants outside these categories (answers such as “do not know” or “prefer not to answer”) were labelled as “Unknown.”

## **Visit type**

OCT measurements were obtained either at the baseline assessment visit or at the subsequent repeat assessment. To account for potential systematic differences in retinal thickness across visits, we included visit type (baseline vs. repeat) as a covariate in regression models. Baseline visits were used as the reference category.

## Education

Educational attainment was transformed into a continuous measure as the age at which full-time education was completed. We first used Field 6138 (highest qualification achieved) and mapped each category to the typical age of completion of full-time education in the UK:

- Degree = 21 years
- A/AS levels = 18 years
- GCSE/O-levels = 16 years
- CSE = 16 years
- NVQ/HND/HNC = 19 years
- Other professional qualifications = 19 years
- None = 15 years

Years of education were calculated using an algorithm that mapped the highest qualification (Field 6138) to standard completion ages. For participants with missing data in Field 6138, completion age was imputed from Field 845 (questionnaire “age completed full-time education”), excluding values outside the plausible range (<10 or >40 years).

## Townsend Deprivation Index

Area-level deprivation was measured using the Townsend Deprivation Index (UK Biobank Data-Field 22189), derived from national census data and linked to participants’ residential postcode at recruitment. The Townsend Index is a continuous score based on four components: unemployment, non-car ownership, non-home ownership, and household overcrowding. Higher values indicate greater material deprivation.^7^

Air pollution
Exposure to ambient air pollution was quantified using annual average concentrations of fine particulate matter (PM₂.₅), expressed as particles with an aerodynamic diameter ≤2.5 μm. For our analyses, PM₂.₅ levels were extracted from UK Biobank data (Field 24006) and used as a continuous variable.

Diabetes

Diabetes mellitus status was ascertained from two complementary sources within UK Biobank. Primary case identification was based on hospital admission records using ICD-9 and ICD-10 codes (ICD-9: 2500–2509; ICD-10: E10–E14). To maximise case detection and capture participants not recorded in hospital data, we also incorporated self-reported information from the touchscreen **questionnaire** (Field 2443: “Diabetes diagnosed by a doctor”). Questionnaire responses were aligned to the visit used for OCT–SRT pairing (baseline or repeat), with fallback to other available instances if missing. Participants were classified as diabetic if either source indicated a diagnosis.

## Body mass index

Body mass index was derived from Data-Field 21001 (UK Biobank), expressed in kg/m². For each participant, BMI was aligned with the visit used for pairing of OCT and hearing measures (baseline or repeat assessment). When unavailable, values from subsequent assessment instances (2_0, 3_0) were used as fallback. This approach ensured temporal consistency of BMI with the main study variables while maximizing data completeness.

Alcohol consumption
Alcohol consumption was assessed through the baseline UK Biobank questionnaire (Data-Field 1558 and related beverage-specific intake questions). Responses were converted into standardized ethanol intake (g/week) using UK Biobank dietary methodology as previously described (Stuart et al., Ophthalmology Glaucoma 2023).^8^ Implausible values were excluded, and the variable was analysed as a continuous measure of total alcohol consumption.

## Smoking status

Smoking status was derived from UK Biobank Data-Field 20116, which records whether a participant reports being a current, previous, or never smoker. Responses were available across multiple assessments (baseline and repeat visits). For each participant, we aligned smoking status with the OCT/SRT assessment visit (baseline or repeat). When the corresponding assessment was missing, we used data from the closest available visit. Smoking status was categorized as never*,* previous*,* or current smoker, excluding responses coded as “do not know” or “prefer not to answer”.

## Spherical equivalent

Spherical equivalent values were derived from UK Biobank autorefractor data using the standard formula (sphere + 0.5 × cylinder), calculated separately for each of up to three measurements per eye. For both baseline (instance 0) and first repeat assessment (instance 1), these replicate measures were averaged to generate right-eye (SER) and left-eye (SEL) means, and an overall visit-specific mean SE (SE₁ for baseline, SE₂ for repeat). Only measurements flagged as reliable by UK Biobank quality indicators (fields 5189, 5191) were retained. Participants with evidence of prior refractive surgery or retinal procedures (fields 5324, 5325, 5328) were excluded. Low myopes (–1 to –3 D) were additionally excluded if biometric parameters were inconsistent (fields 5441, 2207, 2217). Cases showing marked interocular discordance were removed, including: (i) eyes of opposite sign (one myopic ≤ –1 D and the other hyperopic ≥ +1 D), (ii) high ametropia in one eye (≤ –6 D or ≥ +3 D) with the fellow eye near emmetropia (–1 to +1 D), or (iii) large anisometropia (≥ 10 D) in either direction. The final composite SE variable paired each participant to their chosen OCT/SRT visit (baseline or repeat), taking the corresponding SE value if available; if that visit’s SE was missing but the other visit’s SE was present, the latter was used as a fallback. Although SE was not always recorded at the exact same visit as OCT and hearing assessment, refractive error is highly stable in middle-aged and older adults, and this approach maximised sample size while preserving measurement reliability.

## Systemic systolic blood pressure

Systolic (SBP) was obtained from automated readings recorded at the UK Biobank assessment centres (Data-Field 4080). At each assessment, two measurements were taken and averaged to obtain a single SBP value per visit. For each participant, blood pressure values were aligned with the visit used for OCT and SRT pairing (baseline or first repeat assessment). When the value at the paired visit was missing, the alternative visit was used as a fallback. Implausible values were excluded (<70 or >270 mmHg).

# Statistical analysis

## Outcomes and main exposure

We investigated the relationship between hearing function and retinal thickness using multivariable linear regression models. Each model included one OCT-derived retinal thickness measure (in microns or standardized z-scores) as the dependent variable and SRT as the main exposure. Analyses were conducted separately for nine OCT-derived measures, corresponding to individual or composite layers defined by the UK Biobank segmentation protocol, as described in the previous paragraph “Retinal thickness assessment”.

**SRT** was analysed in three complementary ways:

1. As a continuous variable in native units (measured in dB).
2. As a standardised variable: SRT and retinal thickness expressed as z-scores, allowing direct comparison of effect sizes across retinal layers.
3. As a categorical variable, with hearing status dichotomised as normal (≤ –5.5 dB) vs impaired (> –5.5 dB), where impaired included both insufficient (–5.5 to –3.5 dB) and poor (≥ –3.5 dB) hearing. Normal hearing served as the reference category.

## Regression models

Three sets of models were fitted for each OCT-defined measure:

1. Continuous model: SRT (dB) and retinal thickness (µm) were analysed in their original scales. For this analysis, two models were specified: model 1, adjusted for fixed, non-modifiable covariables (age, sex, SE, ethnicity, and visit type), and model 2, which additionally included modifiable risk factors (education, Townsend deprivation index, PM₂.₅ exposure, alcohol intake, diabetes, SBP, smoking, and BMI).
2. Standardised model: SRT and retinal thickness values were standardised (z-scores) to allow comparison of effect sizes across retinal layers, using model 2. This analysis was used to generate the forest plot and the supplementary comparison tables.
3. Categorical model: SRT was entered as a categorical predictor, with hearing performance defined according to UK Biobank thresholds: normal (≤ –5.5 dB), insufficient (–5.5 to –3.5 dB), and poor (≥ –3.5 dB). To ensure statistical stability, the insufficient and poor groups were combined into a single impaired hearing category and compared with the normal group. Model 2 was applied. Sensitivity analyses considering the three groups separately are reported in the Supplementary Material.

For categorical covariates, the following reference categories were used: baseline visit (vs. repeat), male sex (vs. female), White ethnicity (vs. other ethnic groups), never smoked (vs. former/current), and no diabetes (vs. diabetes).

To evaluate whether the SRT–retina thickness association departed from linearity, we examined scatter-density plots of covariate-adjusted residuals (model 2), which showed no curvilinear patterns. We additionally compared linear models with spline-based specifications and with alternative SRT scales (dB, linear-pressure, and log-linear). None of these approaches materially improved model fit, supporting the use of linear terms for SRT in all analyses.

## Interpretation

To improve interpretability, regression coefficients for SRT were expressed as “age-equivalent cross-sectional changes”. For each model, the SRT coefficient ($\beta_{\left\{ SRT \right\}}$) was divided by the corresponding age coefficient (${|\beta}_{\left\{ age \right\}}|$) yielding the estimated number of years of age-equivalent difference that produced an equivalent effect on retinal thickness:

$$Age-equivalent difference (years)=\frac{\beta_{\left\{ SRT \right\}}}{{|\beta}_{\left\{ age \right\}}|}$$

Where $\beta_{\left\{ SRT \right\}}$ corresponds to the change in retinal thickness (in µm) per 1 dB increase of SRT in the continuous model, or the difference in thickness between hearing groups (impaired vs normal, hearing) in the categorical model); ${|\beta}_{\left\{ age \right\}}|$ represents the corresponding change in retinal thickness per year of age-equivalent difference, expressed in the same unit (microns).

## Effect modification by age

To examine whether the association between hearing performance and retinal thickness varied by age, we conducted secondary analyses using both interaction models and age-stratified models.

### Interaction models

For each retinal layer $Y$, we fitted an extended version of model 2 (maximally adjusted) including a continuous interaction term between continuous SRT and age:

$$Y=\beta_{0}+\beta_{1}\mathrm{SRT}+\beta_{2}\mathrm{Age}+\beta_{3}(\mathrm{SRT}\times\mathrm{Age})+\sum_{k} \gamma_{k}C_{k}+\varepsilon,$$

where SRT is expressed in dB, age in years, and $C_{k}$are the covariates included in model 2 (sex, ethnicity group, SE, education, Townsend deprivation index, PM_2.5_ exposure, alcohol consumption, diabetes, SBP, smoking status, BMI, and visit type).
The parameter $\beta_{3}$ quantifies the age-dependent change in the SRT–retinal thickness association. Wald tests for $\beta_{3}=0$ were used as formal tests of effect modification.

### Age-stratified models

To provide age-specific effect estimates and facilitate interpretation of the interaction results, we repeated Model 2 within two predefined age strata. We first inspected the age distribution and derived age quartiles (Q1: 40–50, Q2: 51–58, Q3: 59–63, Q4: 64–75 years); for analytical stability and to maximise contrast between mid-life and older age, the lower two quartiles (40–58 years) and upper two quartiles (59–75 years) were combined to form two age groups of comparable size.

Within each age group, the following model was fitted:

$$Y=\beta_{0}+\beta_{1}\text{ }\mathrm{SRT}+\sum_{k} \gamma_{k}C_{k}+\varepsilon,$$

using the same covariate set $C_{k}$and the same reference categories as in Model 2.
These models quantify the magnitude of the SRT–retinal thickness association separately in mid-life and older participants.

## Visual acuity analysis

Visual acuity (VA) analyses were restricted to participants who met all hearing-related exclusion criteria (including ototoxic medication use, ear surgery, non-sensorineural hearing disorders, excessive noise exposure, mental or neurological disorders, and extreme SRT values), had available VA measurements. VA was assessed under habitual correction, with participants asked to wear their usual distance glasses or contact lenses if available. Participants who normally required distance correction but did not have it available at the assessment visit were excluded. VA measurements were aligned with the same visit used for hearing and OCT data (baseline when available, otherwise the earliest follow-up visit).

Mean VA (logMAR) and standard deviation were calculated separately for participants with normal hearing and impaired hearing, defined according to the hearing classification used in the main analyses.

To evaluate the association between hearing status and VA while accounting for potential confounders, multivariable linear regression models were fitted with VA as the dependent variable and hearing status (impaired vs normal) as the main exposure. Models were adjusted for age, sex, ethnicity, visit type (baseline vs repeat), spherical equivalent, education, Townsend deprivation index, PM₂.₅ exposure, alcohol intake, diabetes, systolic blood pressure, smoking status, and BMI.

## Sensitivity analyses

Three sensitivity analyses were performed to evaluate the robustness of our findings.

## 1. Extreme SRT values.

We repeated the analyses including participants with extreme SRT values (> 5 dB), who had been excluded from the main analysis as likely outliers using the native units model. The extended dataset was analysed using the same model specifications as in the main analysis (minimally and fully adjusted models). Formal interaction tests were performed by including a binary indicator for extreme SRT status (≤ 5 dB vs > 5 dB) and its interaction with the continuous SRT variable in the regression model; regression coefficients and p-values were compared to those from the main analysis to test whether the SRT–retinal thickness association differed between participants with typical and extreme SRT values.

### 2. Original three-category hearing variable.

We repeated the categorical analyses using the original three hearing categories (normal, insufficient, poor) instead of the collapsed impaired vs normal grouping, to assess consistency across finer hearing subgroups.

### 3. Exclusion of retinal/ocular diseases.

To assess whether our findings could be influenced by underlying ocular pathology, we repeated the analyses after removing participants with any of the following diagnoses, identified through UK Biobank ICD-9 and ICD-10 codes:

- Glaucoma: ICD-9 365.0-365.9; ICD-10 H40.*, H42.*.
- Age-related macular degeneration (AMD): ICD-9 362.50-362.52; ICD-10 H35.3*
- Diabetic retinopathy: ICD-9 362.0*; ICD-10 E10.3*, E11.3*, E12.3*, E13.3*, E14.3*, H36.0*.
- Retinal vascular disease: ICD-9 362.3; ICD-10 H34.*, H35.0

All participants with at least one relevant code were excluded. Analyses were rerun using the maximally adjusted (model 2) specification in native units.

## Statistical reporting

We reported β coefficients, 95% confidence intervals, and p-values. Statistical significance was defined as two-sided p < 0.05. As separate regression models were fitted for each of the nine retinal layers, Bonferroni correction was applied to account for multiple comparisons within each analysis family (native units, standardised, and categorical models). The adjusted significance threshold was set at p < 0.0056 (0.05/9). Both nominal and Bonferroni-corrected significance are reported, with results remaining significant after correction indicated by an asterisk (*). Forest plots were generated to visualize standardised associations across retinal layers.

## Statistical software

Analyses were performed in R (version 4.4.2) using RStudio (version 2024.12.0, Posit Software, PBC) and dplyr, broom, purrr, tibble, stringr and ggplot2 packages.

# References

1.	Hall SJ. The development of a new English sentence in noise test and an English number recognition test. Published online 2006.

2.	Smits C, Kapteyn TS, Houtgast T. Development and validation of an automatic speech-in-noise screening test by telephone. *Int J Audiol*. 2004;43(1):15-28. doi:10.1080/14992020400050004

3.	Vlaming MSMG, Kollmeier B, Dreschler WA, et al. Hearcom: Hearing in the communication society. *Acta Acust united with Acust*. 2011;97(2):175-192. doi:10.3813/AAA.918397

4.	Normative data for the Digit Triplet Test, UK Biobank Resource 138440. Accessed September 15, 2025. https://biobank.ctsu.ox.ac.uk/ukb/refer.cgi?id=138440

5.	Keane PA, Grossi CM, Foster PJ, et al. Optical Coherence Tomography in the UK Biobank Study – Rapid Automated Analysis of Retinal Thickness for Large Population-Based Studies. *PLoS One*. 2016;11(10):e0164095. doi:10.1371/journal.pone.0164095

6.	Yang Q, Reisman CA, Wang Z, et al. Automated layer segmentation of macular OCT images using dual-scale gradient information. *Opt Express*. 2010;18(20):21293. doi:10.1364/OE.18.021293

7.	2011 UK Townsend Deprivation Scores - Dataset - UK Data Service CKAN. Accessed October 7, 2025. https://statistics.ukdataservice.ac.uk/dataset/2011-uk-townsend-deprivation-scores

8.	Stuart K V., Luben RN, Warwick AN, et al. The Association of Alcohol Consumption with Glaucoma and Related Traits: Findings from the UK Biobank. *Ophthalmol Glaucoma*. 2023;6(4):366-379. doi:10.1016/j.ogla.2022.11.008
