# Supplementary material for: Association Between Retinal Layer Thickness and Hearing Performance in the UK Biobank: A Cross-Sectional Study
Source: Invest Ophthalmol Vis Sci. 2026 Nov 4;67(13):10. doi: 10.1167/iovs.67.13.10 (PMC13743836; doi:10.1167/iovs.67.13.10)
Supplement: Supplementary file 2 [file iovs-67-13-10_s002.docx]

**Supplementary Table 5.** OCT-derived retinal thickness measures (µm) of eligible UK Biobank participants.

| Retinal layer | Mean (SD) |
| --- | --- |
| Macula | 277.62 (11.67) |
| RPE | 25.04 (2.52) |
| RNFL | 29.62 (4.54) |
| INL | 32.48 (2.06) |
| GCIPL | 73.14 (5.96) |
| INL–ELM | 80.37 (5.62) |
| ELM–ISOS | 23.59 (1.32) |
| ISOS–RPE | 38.42 (3.35) |
| INL–RPE | 142.37 (6.86) |

**Supplementary Table 6.** Fully adjusted linear regression results for the association between standardized SRT (z-score) and retinal layer thickness (z-score).

| ****Retinal Layer**** | ****Estimate (β)**** | ****Std. Error**** | ****p-value**** | ****95% CI lower**** | ****95% CI upper**** |
| --- | --- | --- | --- | --- | --- |
| RNFL | −0.0031 | 0.0048 | 0.525 | −0.0125 | 0.0064 |
| **GCIPL** | **−0.0204** | **0.0046** | **1.08×10⁻⁵** | **−0.0295** | **−0.0113** |
| INL | **−0.0183** | **0.0055** | **9.73×10⁻⁴** | **−0.0291** | **−0.0074** |
| INL–ELM | **−0.0166** | **0.0056** | **3.20×10⁻³** | **−0.0276** | **−0.0055** |
| INL–RPE | **−0.0148** | **0.0056** | **8.85×10⁻³** | **−0.0259** | **−0.0037** |
| ELM–ISOS | 0.0038 | 0.0056 | 0.496 | −0.0071 | 0.0147 |
| ISOS–RPE | −0.0043 | 0.0057 | 0.457 | −0.0155 | 0.0070 |
| RPE | 0.0077 | 0.0056 | 0.172 | −0.0033 | 0.0187 |
| ****Macula total**** | **−0.0236** | **0.0056** | **2.51×10⁻⁵** | **−0.0345** | **−0.0126** |

Estimates represent the SD change in retinal thickness per 1 SD worse SRT. Models were adjusted for age, sex, spherical equivalent, ethnicity, visit type, education, Townsend deprivation index, PM₂.₅ exposure, alcohol intake, diabetes, systolic blood pressure, smoking status, and body mass index. Statistically significant results (p < 0.05) are shown in bold.
