# Supplementary material for: Association Between Retinal Layer Thickness and Hearing Performance in the UK Biobank: A Cross-Sectional Study
Source: Invest Ophthalmol Vis Sci. 2026 Nov 4;67(13):10. doi: 10.1167/iovs.67.13.10 (PMC13743836; doi:10.1167/iovs.67.13.10)
Supplement: Supplementary file 3 [file iovs-67-13-10_s003.docx]

**Supplementary Results**

# Age-related variation in the SRT–retinal thickness association

## Interaction analysis

In models including an SRT×age interaction term (**Supplementary Table 7**), we found clear evidence of age-related effect modification for for GCIPL (p = 0.0007), INL (p = 0.0024), ISOS–RPE (p = 0.041), and total macular thickness (p = 0.010), with stronger inverse SRT–thickness associations at older ages. In contrast, the interaction term was positive for ELM–ISOS (p = 0.0054), indicating a modest attenuation of the association in older adults. No significant age-related variation was observed for RNFL, INL–ELM, INL–RPE, or RPE. Overall, these findings suggest that age amplifies the inverse SRT–thickness relationship predominantly in inner and photoreceptor layers, whereas outer retinal composites and RPE remain largely age-stable.

**Supplementary Table 7.** Interaction between SRT and age for each retinal layer.

| **Retinal Layer** | **β (SRT×Age)** | **p-value** |
| --- | --- | --- |
| RNFL | –0.000076 | 0.966 |
| **GCIPL** | **–0.007706** | **0.0007** |
| **INL** | **–0.002886** | **0.0024** |
| INL–ELM | –0.001840 | 0.483 |
| INL–RPE | –0.003338 | 0.300 |
| **ELM–ISOS** | **+0.001689** | **0.0054** |
| **ISOS–RPE** | **–0.003255** | **0.041** |
| RPE | +0.000166 | 0.888 |
| **Macula total** | **–0.013912** | **0.0104** |

β values represent the regression coefficient for the SRT×age interaction term from maximally adjusted linear models (Model 2), indicating whether the association between poorer hearing (higher SRT) and retinal thickness varies with age. Negative coefficients indicate a stronger inverse SRT–thickness association at older ages, whereas positive coefficients indicate attenuation of the association with age. P-values refer to nominal two-sided significance tests of the interaction term.

## Age-stratified models

Age-stratified analyses compared participants in the lower two age quartiles (40–58 years; n = 16,918), with those in the upper two age quartiles (59–75 years; n = 15,695). Within each stratum, we reestimated the association between SRT and retinal thickness using the maximally adjusted model (model 2). Inverse associations between SRT and retinal thickness were broadly consistent across both age groups, with slightly stronger associations for GCIPL and INL in older adults. Macular thickness showed similar inverse associations in both strata. Full results are shown in **Supplementary Figure 2** and **Supplementary Table 8**.

**Supplementary Figure 2.** Forest plot: age-stratified associations between SRT and retinal layer thickness

Association between poorer hearing (higher SRT) and retinal layer thickness within two age strata: 40–58 years (Q1–Q2) and 59–75 years (Q3–Q4). Points represent β coefficients (µm change in thickness per 1 dB increase in SRT), and horizontal bars indicate 95% confidence intervals. Models are adjusted for age (within strata), sex, SE, ethnicity, education, Townsend deprivation index, PM2.5, alcohol intake, diabetes, SBP, smoking, BMI, and visit type.

**Supplementary Table 8.** Age-stratified association between SRT and retinal thickness.

| **Retinal layer** | **Age group** | **β (95% CI)** | **p-value** |
| --- | --- | --- | --- |
| **RNFL** | 40–58 | –0.014 (–0.059 to 0.030) | 0.52 |
|  | 59–75 | –0.006 (–0.047 to 0.036) | 0.79 |
| **GCIPL** | **40–58** | **–0.060 (–0.117 to –0.004)** | **0.037** |
|  | **59–75** | **–0.084 (–0.136 to –0.032)** | **0.0016** |
| **INL** | 40–58 | –0.017 (–0.040 to 0.007) | 0.17 |
|  | **59–75** | **–0.027 (–0.048 to –0.005)** | **0.015** |
| **INL–ELM** | 40–58 | –0.064 (–0.130 to 0.001) | 0.055 |
|  | 59–75 | –0.049 (–0.109 to 0.011) | 0.11 |
| **INL–RPE** | 40–58 | –0.071 (–0.153 to 0.011) | 0.088 |
|  | 59–75 | –0.038 (–0.110 to 0.034) | 0.31 |
| **ELM–ISOS** | 40–58 | –0.0015 (–0.017 to 0.014) | 0.85 |
|  | 59–75 | +0.0040 (–0.010 to 0.018) | 0.57 |
| **ISOS–RPE** | 40–58 | –0.0069 (–0.049 to 0.035) | 0.75 |
|  | 59–75 | +0.0074 (–0.027 to 0.041) | 0.67 |
| **RPE** | 40–58 | +0.016 (–0.015 to 0.048) | 0.31 |
|  | 59–75 | +0.008 (–0.017 to 0.033) | 0.52 |
| **Macular total** | **40–58** | **–0.167 (–0.303 to –0.032)** | **0.0157** |
|  | **59–75** | **–0.152 (–0.276 to –0.029)** | **0.0156** |

Maximally adjusted age-stratified associations between speech reception threshold (SRT, per 1 dB higher) and retinal thickness across participants aged 40–58 and 59–75 years.

# Visual acuity

After applying all hearing-related exclusion criteria and restricting the analysis to participants with available VA measurements and complete covariate data, 45,967 participants were eligible for inclusion in the VA analyses. Of these, 2,361 participants were excluded because habitual distance correction was recorded as unavailable at the assessment visit. The final analytical sample for VA therefore comprised 43,606 participants, of whom 35,795 had normal hearing and 7,811 had impaired hearing.

Participants with impaired hearing had worse VA compared with those with normal hearing. Mean (SD) VA was –0.002 (0.16) logMAR in participants with normal hearing and 0.035 (0.16) logMAR in those with impaired hearing. In univariable analysis, impaired hearing was associated with significantly worse VA (Welch’s t-test, p < 2.2 × 10⁻¹⁶).

In the maximally adjusted multivariable linear regression model, impaired hearing remained independently associated with worse VA. Compared with participants with normal hearing, those with impaired hearing had a 0.015 logMAR worse VA (β = 0.0147; 95% CI 0.011 to 0.019; p = 1.15 × 10⁻¹³).

# Sensitivity analysis

To test the robustness of our findings, we first repeated the analyses including participants with extreme SRT values (>5 dB). Of the 55 participants excluded at this step in the main analysis, 41 had complete covariate data and were included in the sensitivity analysis. Effect estimates remained similar in magnitude and direction, and the pattern of statistical significance across retinal layers was unchanged.

We next examined insufficient (n=4,858) and poor (n = 617) hearing groups separately; results broadly mirrored the main impaired vs normal analysis, but estimates in the poor hearing group were less precise because of the smaller sample size. Associations for GCIPL and macular thickness remained statistically significant both in the poor and insufficient hearing group after applying Bonferroni correction for multiple comparisons (p < 0.0056), whereas INL, INL–ELM, INL–RPE remained significant only in the insufficient hearing group and RNFL only in the poor hearing group. Overall, the results for the poor hearing category were characterised by wide confidence intervals and did not follow a consistent dose–response pattern, whereby more severe hearing loss would be expected to correspond to lower retinal thickness. This pattern suggests that the poor group lacked sufficient statistical power to yield coherent results, supporting the use of a combined impaired category in the main analyses. Results for the three hearing categories are presented in **Supplementary Table 9**.

**Supplementary Table 9.** Association between insufficient and poor hearing group compared to the normal hearing group.

| **Retinal Layer** | **SRT Group** | **β (95% CI)** | **p-value** | **Age-equivalent difference (years)** |
| --- | --- | --- | --- | --- |
| **RNFL** | Insufficient | −0.03 (−0.15, 0.08) | 0.58 | −1.04 years |
|  | **Poor** | **−0.31 (−0.61, −0.01)** | **0.04** | **−9.87 years** |
| **GCIPL** | **Insufficient** | **−0.29 (−0.43, −0.14)** | **1.3×10⁻⁴ *** | **−2.06 years** |
|  | **Poor** | **−0.51 (−0.89, −0.13)** | **0.008** | **−3.69 years** |
| **INL** | **Insufficient** | **−0.10 (−0.16, −0.04)** | **0.001 *** | **−2.62 years** |
|  | Poor | −0.11 (−0.27, 0.05) | 0.18 | −2.84 years |
| **INL–ELM** | **Insufficient** | **−0.25 (−0.42, −0.08)** | **0.004 *** | **−12.01 years** |
|  | Poor | −0.17 (−0.61, 0.26) | 0.44 | −8.43 years |
| **INL–RPE** | **Insufficient** | **−0.28 (−0.49, −0.07)** | **0.008** | **−6.12 years** |
|  | Poor | −0.43 (−0.96, 0.11) | 0.12 | −9.28 years |
| **ELM–ISOS** | Insufficient | −0.01 (−0.05, 0.03) | 0.55 | −0.40 years |
|  | Poor | +0.01 (−0.09, 0.12) | 0.79 | +0.45 years |
| **ISOS–RPE** | Insufficient | −0.02 (−0.13, 0.08) | 0.66 | −3.42 years |
|  | Poor | −0.26 (−0.52, 0.01) | 0.059 | −37.59 years |
| **RPE** | Insufficient | +0.05 (−0.03, 0.13) | 0.21 | +1.01 years |
|  | Poor | +0.07 (−0.13, 0.27) | 0.49 | +1.43 years |
| **Macula total** | **Insufficient** | **−0.72 (−1.07, −0.37)** | **6.0×10⁻⁵ *** | **−2.81 years** |
|  | **Poor** | **−1.36 (−2.27, −0.46)** | **0.003 *** | **−5.34 years** |

Maximally adjusted multivariable regression models (model 2) with “Normal hearing” as reference. Results are expressed as regression coefficients (β, µm), 95% confidence intervals, p-values, and age-equivalent difference expressed in years. Negative values indicate thinner retinal layers with worse hearing. Significant associations (p < 0.05) are shown in bold. p-values below the Bonferroni-adjusted significance threshold (p < 0.0056) are indicated with an asterisk (*).

Finally, results were unchanged when excluding all participants with AMD, glaucoma, diabetic retinopathy or other retinal vascular disease (n = 690, 534, 196, and 116, respectively). Effect estimates, direction, and layer-specific patterns were nearly identical to the main fully adjusted models, with complete overlap of confidence intervals, confirming that underlying ocular pathology did not drive the observed associations (**Supplementary Table 10**).

**Supplementary Table 10.** Sensitivity analysis excluding AMD, glaucoma, diabetic retinopathy and retinal vascular disease.

| **Retinal Layer** | **β (95% CI)** | **p-value** |
| --- | --- | --- |
| RNFL | −0.0056 (−0.0368 to +0.0255) | 7.23×10⁻¹ |
| **GCIPL** | −0.0885 (−0.1281 to −0.0489) | 1.20×10⁻⁵ |
| **INL** | −0.0282 (−0.0448 to −0.0116) | 8.53×10⁻⁴ |
| **INL–ELM** | −0.0739 (−0.1197 to −0.0280) | 1.60×10⁻³ |
| **INL–RPE** | −0.0877 (−0.1441 to −0.0314) | 2.28×10⁻³ |
| ELM–ISOS | +0.0019 (−0.0087 to +0.0125) | 7.24×10⁻¹ |
| ISOS–RPE | −0.0176 (−0.0455 to +0.0103) | 2.16×10⁻¹ |
| RPE | +0.0142 (−0.0065 to +0.0349) | 1.79×10⁻¹ |
| **Macula total** | −0.2133 (−0.3082 to −0.1185) | 1.05×10⁻^5^ |

Maximally adjusted linear regression models (model 2) examining the association between speech reception threshold (SRT, dB) and retinal layer thickness (µm), after excluding all participants with glaucoma, age-related macular degeneration (AMD), diabetic retinopathy or retinal vascular disease based on ICD-9/10 coding. Results are reported as β coefficients (µm per 1 dB higher SRT) with 95% confidence intervals and p-values.
